# Inhibiting RIPK1 Limits Neuroinflammation and Alleviates Postoperative Cognitive Impairments in D-Galactose-Induced Aged Mice

**DOI:** 10.3389/fnbeh.2018.00138

**Published:** 2018-07-10

**Authors:** Shangchun Duan, Xueqin Wang, Gong Chen, Chengxuan Quan, Shuangquan Qu, Jianbin Tong

**Affiliations:** ^1^Department of Anesthesiology, The Third Xiangya Hospital, Central South University, Changsha, China; ^2^Center for Experimental Medicine, The Third Xiangya Hospital, Central South University, Changsha, China; ^3^Department of Anesthesiology, Hunan Children’s Hospital, Changsha, China

**Keywords:** surgery, cognitive deficit, neuroinflammation, necrostatin-1, elderly

## Abstract

Neuroinflammation plays a critical role in the pathogenesis of postoperative cognitive dysfunction (POCD) of the elderly patients. Receptor-interacting protein kinase1 (RIPK1) is a key molecular switch modulating inflammation, apoptosis and necroptosis. Here, we investigated whether inhibiting RIPK1 by necrostatin-1 (Nec-1) could limit neuroinflammation and attenuate POCD in D-Galactose (D-Gal)-induced aged mice. The mice were subjected to anesthesia and partial hepatectomy, and necrostatin-1 was administered intraperitoneally 1 h prior to anesthesia and surgery. Cognitive function and movement were tested 24 h after surgery by open field, Barnes maze and puzzle box. The hippocampal tissues were collected to detect the following: neuroinflammation (Iba-1, IL-1α, IL-1β, TNF-α), Necroptosis (Propidium Iodide (PI) labeling, RIPK1, nuclear transcription factor kappa B (NF-κB) and neuroplasticity (doublecortin (DCX), NR2B, GluA1, GluA2). We found that anesthesia and surgery induced a significant deficit in spatial memory acquisition and impairment of executive function and memory to simple task in D-Galactose-induced aged mice. Inhibiting RIPK1 by necrostatin-1 strikingly mitigated cognitive impairment and alleviated postoperative amplified neuroinflammation, necroptosis and GluA1 loss in hippocampus. These suggest that targeting RIPK1 by necrostatin-1 may serve as a promising therapeutics for prevention of POCD in elderly patients.

## Introduction

Postoperative cognitive dysfunction (POCD) is a common postoperative complication in elderly patients, characterized by the impairment of memory, information processing ability, and mental flexibility. Neuroinflammation plays a critical role in the pathogenesis of POCD (Skvarc et al., 2018). Preclinic studies have shown that peripheral surgery or anesthesia alone both can activate microglia and increase the levels of inflammatory factors in brain (Vutskits and Xie, 2016; Zhang et al., 2016). Intracisternal anti-inflammation treatments could obviously inhibit neuroinflammation and prevented POCD (Barrientos et al., 2012; Ma et al., 2015; Li et al., 2017). These suggest that limiting neuroinflammation during perioperative period is a possible way to prevent or alleviate POCD. Due to the difficulty of intracisternal administration in patients with surgery, people had tried to limit perioperative neuroinflammation by systemic inflammatory factor neutralization with specific antibodies, and had detected efficacious inhibition of neuroinflammation and significant improvement of postoperative cognitive function (Terrando et al., 2010, 2016). However, specific antibodies of inflammatory factors are expensive, and possibly impact wound healing by blocking the peripheral inflammatory response. Thus, new treatment of limiting perioperative neuroinflammation is still pressing for the prevention of POCD.

Receptor-interacting protein kinase 1 (RIPK1) is a key molecule of necroptosis. It can modulate the intracellular signaling in response to stimuli such as TNF and ligands of Toll-like receptors (Pasparakis and Vandenabeele, 2015; Weinlich et al., 2017). Especially, it plays an important role in nuclear transcription factor kappa B (NF-κB)-dependent inflammatory response, caspase-8-dependent apoptosis and mixed lineage kinase like (MLKL)-dependent necroptosis (Pasparakis and Vandenabeele, 2015; Weinlich et al., 2017). Previous studies have shown that inhibiting RIPK1 obviously alleviates neuroinflammation and protects brain function in brain ischemic models (Linkermann et al., 2013). Evidences emerged that RIPK1 activation increased in the microglia of brains in Alzheimer’s disease (Ofengeim et al., 2017; Rubinsztein, 2017). Furthermore, inhibiting RIPK1 activation alleviated neuroinflammation and cognitive impairments during Alzheimer’s disease, and didn’t show obvious toxic effect (Ofengeim et al., 2017; Yang et al., 2017). These suggest that inhibiting RIPK1 could limit neuroinflammation and protect brain function in acute and chronic brain damage. Though POCD is highly related with neuroinflammation, whether inhibiting RIPK1 can prevent POCD remain unknown.

D-Galactose (D-Gal) can induce the aged phenotype in mice (Shwe et al., 2017). And the occurrence of POCD is aging-dependent (Le et al., 2014). Thus, we here induced aged mice via intraperitoneal injection of D-Gal for 2 months and detected the effects of inhibiting RIPK1 by necrostatin-1 (Nec-1; Yang et al., 2017) on POCD in the aged mice. We found that inhibiting RIPK1 by Nec-1 significantly mitigated postoperative cognitive deficits and alleviated perioperative neuroinflammation in aged mice. Meanwhile, inhibiting RIPK1 by Nec-1 also rescued surgery-induced changes in synaptic GluA1 loss and necroptotic cell death. These suggest that targeting RIPK1 may be a promising method for prevention of POCD.

## Materials and Methods

### Animals

Eighty-eight healthy 8-week-old male C57BL/6J mice weighing 20–25 g were purchased from the experimental animal center of Central South University (China). The mice were accessed water and food *ad libitum* at an adequate temperature of 23°C and a relative humidity of 50%–60% with a 12 h light-dark cycle (light on from 7:00–19:00). All the animal experiments were conducted in accordance with the National Institute of Health Guide for the Care and Use of Laboratory Animals (NIH Publications No. 80-23) revised 1996 and approved by the Animal Ethics Committee of the third Xiangya Hospital (Changsha, China, No.: LLSC (LA) 2017-001). All the experiments were made to minimize the number of animals used and their suffering. In order to avoid the subjective bias, the experimenters were blinded to the treatment of the animals and the data statistics.

### D-Gal-Induced Accelerated Aging Model

Following 4 days acclimation to laboratory conditions, 88 mice were administered with 25 mg/d D-Gal (Sigma-Aldrich Co., St. Louis, MO, USA) by intraperitoneal injection as previously reported (Liao et al., 2016), once daily for 2 months to induce accelerated aging model. It was dissolved in 0.9% saline, and injected daily at about 14:00 o’clock during the experimental period. Three mice died from improper intraperitoneal injection of D-Gal in the initial period.

### Grouping of Animals

D-Gal-induced aged mice were randomly divided into three groups, control group (*n* = 19), surgery+DMSO group (Sur+DMSO, *n* = 33), and surgery+Nec-1 group (Sur+Nec1, *n* = 33). The last two groups received anesthesia and left partial hepatectomy. Besides this, the mice in Sur+Nec1 group was pretreated with Nec-1 at 1 h prior to anesthesia and surgery, while the mice in Sur+DMSO group received an equal volume and concentration of dimethyl sulfoxide (DMSO, Sigma-Aldrich Co., St. Louis, MO, USA) in the same way as vehicle control.

Twelve mice in each group were randomly selected exclusively for behavior test. The other mice were sacrificed at 6 h, 3 days and 7 days after anesthesia and surgery, respectively (*n* = 7 at each time point per group).

### Nec-1 Administration

Nec-1 (Selleckchem, Houston, TX, USA) was first dissolved in DMSO to a concentration of 25 μg/μL as stock solutions. Then the stock solutions were diluted 40 times with sterile phosphate buffer saline (PBS) before use to make sure that the concentration of DMSO was 2.5% and the concentration of Nec-1 was 0.625 μg/μL. For Sur+Nec1 group, Nec-1 was administered by intraperitoneal injection with a dose of 6.25 mg/kg 1 h prior to anesthesia and surgery (once per mice) as previously reported (Yang et al., 2017; Zhou et al., 2017). The mice in Sur+DMSO group received an equal volume and concentration of DMSO by intraperitoneal injection at the same time.

### Anesthesia and Partial Hepatectomy

Anesthesia and left partial hepatectomy was conducted as described earlier (Tang et al., 2017). The mice were anesthetized with inhalation sevoflurane. Then the mice were rapidly induced with 5% sevoflurane anesthesia with high flow oxygen (5 L/min) via a cone transparent mask near the nose of mice which was linked with a multi-function monitor (Datex-Ohmeda, Helsinki, Finland). After induction, 2% sevoflurane mixed with 80%–85% oxygen was continuously delivered. The anesthesia duration for each mouse was 2 h. The left partial hepatectomy in mice was performed. Briefly, a small transverse skin incision about 2 cm long was made below the xiphoid process, then the superficial fascia, deep fascia, abdominal muscles and peritoneum was dissected. The left lobe of liver was visualized, carefully isolated, and then securely ligated and subsequently removed. Finally, the incision was sutured layer by layer with 5-0 vicryl thread. After hepatectomy, the mice continued to receive the rest of anesthesia. The mice were placed on a heating pad for postoperative recovery, and then returned into their home cages with water and food available *ad libitum*. 2.5% lidocaine and 2.5% prilocaine cream was applied to the skin incision to alleviate the postoperative pain at the end of procedure and every 8 h within 48 h after surgery.

### Behavior Test

**Figure 1:**
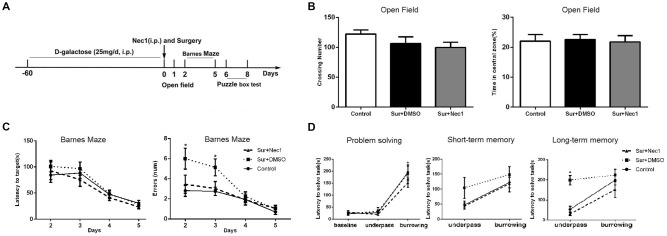
The inhibition of receptor-interacting protein kinase 1 (RIPK1) by necrostatin-1 (Nec-1) attenuates postoperative cognitive deficits in D-Galactose (D-Gal)-induced aged mice. **(A)** The schedule for drug administration, surgery and different behavioral tests. Eight-weeks-old male C57BL/6J mice were intraperitoneally injected with D-Gal (25 mg/d) for 8 weeks. Left partial hepatectomy was conducted under anesthesia. Nec-1 (6.25 mg/kg) or 2.5% dimethyl sulfoxide (DMSO) was injected intraperitoneally prior to surgery. The behavioral tests were performed from 1st day to 8th day after surgery. **(B–D)** Represents behavior results. Data were presented as the mean ± SEM. *n* = 12 in control group, *n* = 12 in Sur+DMSO group and *n* = 10 in Sur+Nec1 group. **(B)** The results of open field test. No obvious difference in crossing number and percentage of time in central zone was detected among three groups (*P* > 0.05 by one-way analysis of variance; ANOVA). **(C)** The results of Barnes maze test for spatial learning and memory. The latency to the target hole was not different among three groups (*P* > 0.05), but the errors were statistical different among three groups (*F* = 5.553, *P* = 0.009); specially, the errors of Sur+DMSO group were significantly more than control and Sur+Nec1 groups on the 3rd day after surgery (*P < 0.05, analyzing by repeated measures ANOVA).** (D)** The results of puzzle box test for executive function. In solving problem, the mice in each group exhibited a longer latency in difficult task (*P < 0.05, analyzing by two-way ANOVA). The mice exposed to surgery exhibited impaired executive function and memory with a longer latency to complete the underpass task (*P < 0.05, analyzing by two-way ANOVA followed by Tukey’s multiple comparison test), but the short-term memory was not affected (*P* > 0.05).

Because two mice in Sur+Nec1 group died from anesthesia accidence before surgery, so finally, the number of each group for behavior test was as follows, 12 for control group, 12 for Sur+DMSO group, and 10 for Sur+Nec1 group. All the tests were conducted in a dim light during the dark cycle. General locomotor activity and anxiety-like behavior was tested with open field test 24 h after surgery, and cognitive performance was assessed with Barnes maze test on postoperative day 2–5, and with puzzle box on postoperative day 6–8 as shown in Figure 1A.

### Open Field Test

Open field test was carried out to assess anxiety-like behavior and locomotive activity as previously described (Zhang et al., 2013). An open-field chamber (width: 50 cm, length: 50 cm, height: 38 cm) which was separated into 25 squares was used. The peripheral zone consisted of 16 squares as identified while the central zone consisted of nine squares. First, the mice were brought to adapt the environment for at least 30 min before the test. Each mouse was placed in the center of chamber with the same direction and allowed free and uninterrupted movement for 5 min. The fecal and urine left by the previous mouse was removed, and 75% ethanol was used to wipe the chamber prior to use and before subsequent tests to remove the remaining scent. The activity of mice was recorded by video suspended above the chamber and the total number of crossing squares and the percentage of crossing into the central zone to the total squares were analyzed.

### Barnes Maze Test

The mice subjected Barnes maze test on postoperative days 2–5, which is usually applied to evaluate the spatial reference memory and learning (Terrando et al., 2016). The apparatus consisted of a circular open platform about 98 cm diameters with 12 holes near the border of the platform with equal size and intervals. And one of these holes was connected with a small dark recessed chamber that called escape box. A 40 watt LED light was suspended right above the center of platform. Barnes maze test were performed during 4 days with three trials per day with an interval of 15 min between each trial. Prior to trial, the mouse was first placed in the center of platform and directly guided to the escape box for habituation to the apparatus. In the subsequent trials and days, the mouse was placed in the center of platform and allowed to freely search the escaped box under the light stimulation for a maximum of 3 min. If failed, it was gently led to the box. Once reaching the escape box, each mouse was allowed to stay for 1 min. During the intervals of each trial, the platform and these holes were cleaned with 75% ethanol. The escape latency (the total time to find the escape box) and the escape errors were recorded and compared. The escape error was judged when the mice lingered beside the other holes except escape hole and its head was extended into the hole and its eyes were inferior to the margin of platform.

### Puzzle Box Test

Puzzle box test is commonly used to evaluate the executive function. It was conducted with three trials per day on day 6–8 after surgery similar to that reported by Zurek et al. (2016). It consisted of an illuminated box (58 × 28 × 27.5 cm^3^) by 40 watt LED light above the center and a dark goal box (14 × 28 × 27.5 cm^3^) separated by a partition. An unobstructed underpass and an open doorway connected the two boxes, which allowed the mice to enter from light to dark compartment. The entrance to the goal box was modified each day to increase the task difficulty. During each trial, the mouse was placed on the center of the light compartment facing away from the doorway, and a maximum time of 5 min was allowed to explore the goal box. If failed, then the mouse was gently directed to the goal box. On day 6, 1st trial, the goal box was accessible freely through the doorway or underpass in 5 min. In 2nd trial, just the underpass could pass to the goal box (problem solving for underpass task) and the mouse was allowed to look for the entrance in 5 min. The short-term and long-term memory for underpass task was tested after 2 min later and on the next day, respectively. On day 7, the difficulty was increased because the doorway was closed and the underpass was covered with saw dust (problem solving for burrowing task). The short-term and long-term memory for burrowing task was tested after 2 min later and on day 8, respectively. During trial eight and nine on day 8 the task became more difficult by obstructing the underpass with a cardboard plug that the mouse had to remove to gain access to the goal box. But unfortunately, the plug task was very difficult for the weak aged mice so that most of them had no interest to complete it. Finally we excluded this plug task. The time spent for each mouse was recorded and analyzed. It was identified successful only when all four paws of the mouse entered the door or underpass.

### Administration of Propidium Iodide (PI)

Propidium iodide (PI; 10 mg/mL, Sigma, St. Louis, MO, USA) labeling was conducted as described previously (Zhang et al., 2014; Kanno et al., 2015) to detect plasma permeability, which is a hallmark of necrotic cell death. Four mice at each time point per group were given PI with a dose of 1 mg/kg dissolved in 0.1 M PB by intraperitoneal injection 30 min prior to sacrifice. The mice were anesthetized with 5% chloral hydrate in 0.9% normal saline, and then perfused transcardially with 0.9% normal saline. The brains were removed out. One half of the brain (*n* = 4) was used for PI labeling and immunohistochemistry, while the hippocampus of the other half was prepared for western blot (*n* = 4) and real-time PCR assay (*n* = 4).

### Tissue Preparation

The brain tissues used for PI labeling and immunostaining were post-fixed in 4% paraformaldehyde for 24 h at 4°C. Then, the brain tissues were placed sequentially in 15% and 30% sucrose in 0.01 M PBS at 4°C. Next, the brain tissues were embedded in Tissue-Tek optimal cutting temperature medium and frozen in liquid nitrogen and stored in −80°C. The brain tissues containing the entirety of hippocampus were serially cut on a freezing sliding microtome (Leica CM1950, Wetzlar, Germany) into 15-μm-thick coronal sections at 150 μm intervals.

For PCR detection and western blot, at the endpoint of experiment, the hippocampal tissues were removed out after deeply anesthesia, and then immediately placed in liquid nitrogen and subsequently stored at −80°C until tissue homogenization.

### Detection of PI-Positive Cells

For detection of PI-labeled cells, brain sections were washed three times in PBS, then cover slipped and mounted with mounting medium with 4′,6-diamidino-2-phenylindole (DAPI, Vector Laboratories Inc., Burlingame, CA, USA) and photographed under a Nikon Eclipse T300 fluorescence microscope (Nikon, Tokyo, Japan). For quantification of PI-positive cells, the photographs were randomly taken from three non-overlapping ×200 fields in each slice. Three sections of each hippocampus were blindly counted and analyzed. PI-positive cells were expressed as PI^+^ cells/× 200 fields.

### Immunohistochemistry

Free-floating sections of hippocampal tissues were washed in PBS, then were sequentially treated with 3% hydrogen peroxide (H_2_O_2_) in 0.01 M PBS for 15 min and 5% bovine serum albumin (BSA, Sigma, St. Louis, MO, USA) in 0.01 M PBS containing 0.3% Triton X-100 for 1 h. Then, they were incubated with rabbit anti-Iba-1 (1:1,000, Wako, Japan) and rabbit anti-DCX (1:1000, Cell Signaling Technology, Danvers, MA, USA) overnight. Then the sections were washed in PBS, subsequently exposed to corresponding biotinylated secondary antibody (1:200, Vector Laboratories, Burlingame, CA, USA). After 1 h incubation with the avidin-biotin complex reagents (ABC Elite Kit, Vector Laboratories, Burlingame, CA, USA), immunoreaction product was visualized by DAB kit (Beijing ZhongshanJinqiao Biological Technology Co., Ltd., China). Finally, the floating sections were mounted, dehydrated, cleared and coverslipped in permount TM mounting medium. The photographs were captured under a microscope (Nikon, Tokyo, Japan) and analyzed. Based on the Iba1 staining, the percentage of activated microglia in dentate gyrus was counted and analyzed as described by Tang et al. (2017).

### Western Blot Analysis

In present study, western blot was used to assess the expression of necroptotic-related protein RIPK1 and NF-κB in the hippocampus. The hippocampal tissues were homogenized in NP40 buffer containing 1% protease inhibitors and 1% phosphatase inhibitor (Sigma-Aldrich Co., St. Louis, MO, USA). Homogenates were centrifuged at 12,000× *g* for 20 min at 4°C. Then the supernatant of hippocampal homogenates were carefully collected. Protein quantification was performed by bicinchoninic acid (BCA) protein assay kit (CWbio, China). The protein samples (40 μg) were separated on a 10% SDS-PAGE gel and then transferred to PVDF membrane (Bio-Rad, Hercules, CA, USA). Non-specific binding were blocked with 5% non-fat milk for 1 h at room temperature. The membrane was incubated with rabbit polyclonal anti-RIP1 (1:200, Cell Signaling Technology, Danvers, MA, USA), or anti-NF-κB antibody (1:1500, Abcam, Cambridge, MA, USA) at 4°C overnight with gentle shaking. The membrane was washed three times for 10 min each with Tris-buffered saline plus 0.3% Tween-20, then incubated with IRDye^®^800CW goat anti-rabbit secondary antibody (1:8000, 926–32211, Li-COR^®^, Lincoln, NE, USA) for 1 h at room temperature. After washed three times, the immunoblotting bands were visualized under Odyssey-CLX infrared imaging systems (Li-COR^®^, Lincoln, NE, USA). GAPDH was used to normalize protein levels as an internal reference control. Integrated density values of specific proteins were quantified using ImageJ software (National Institutes of Health, Bethesda, MD, USA) and the relative expression level of protein were normalized by the ratio of target protein (RIPK1 and NF-κB) to GAPDH.

### Quantitative Real-Time PCR (qPCR) Assay

qPCR assay was to assess the content of critical proinflammatory cytokines TNF-α, IL-α and IL-1β, AMPAR subunit GluA1 and GluA2 as well as NMDAR subunit NR2B in the hippocampus. Total RNA was isolated from homogenized hippocampal tissues using the Trizol extraction method according to the manufacturer’s instructions (Invitrogen, Waltham, MA, USA). The concentration and quality of RNA was evaluated by comparison of optical density value with NanoDrop spectrophotometer (Thermo Scientific, Wilmington, DE, USA). The extracted RNA was converted into cDNA using cDNA Synthesis Kit (GeneCopoeia, Rockville, MD, USA) under GeneAmp^®^PCR system 9700 (Applied Biosystems, Carlsbad, CA, USA). qPCR was performed using mRNA QPCR mix (GeneCopoeia, Rockville, MD, USA) under LightCycler^®^480II analyzer (Roche, Mannheim, Germany). The primers for all assayed genes were determined using reported sequences as listed in Table 1. The qPCR reaction was run at 95°C for 10 min and followed by a repeating 40 cycles of denaturation at 95°C for 10 s, primer annealing at 60°C for 20 s and an extension at 72°C for 20 s, terminated by heating to 72°C for 5 min. After amplification, the gene expression changes were quantified and analyzed using the 2^−ΔΔCt^ method for all samples. The results were normalized to GAPDH as reference gene.

**Table 1:** Primers used for quantitative real-time PCR.

Target gene	Primers	Sequence (5′-3′)
GAPDH	Forward	GGTGAAGGTCGGTGTGAACG
	Reverse	CTCGCTCCTGGAAGATGGTG
NR2B	Forward	GATTCTGCATTGTGAGCCGC
	Reverse	CTCGCTCCTGGAAGATGGTG
GLuA1	Forward	GGACAACTCAAGCGTCCAGA
	Reverse	CTCGCTCCTGGAAGATGGTG
GLuA2	Forward	CCCGGAAGATTGGGTACTGG
	Reverse	ACGCTCATTCCCTTCAAGCA
IL-1α	Forward	CGCTTGAGTCGGCAAAGAAAT
	Reverse	CTTCCCGTTGCTTGACGTTG
IL-1β	Forward	GCCCATCCTCTGTGACTCAT
	Reverse	AGGCCACAGGTATTTTGTCG
TNF-α	Forward	ATGCACCACCATCAAGGACTCAA
	Reverse	ACCACTCTCCCTTTGCAGAACTC

### Statistical Analysis

The data were presented as mean ± standard error (mean ± SEM) and the statistical graphs were processed using GraphPad Prism 5.0 software (GraphPad Software Inc., La Jolla, CA, USA) and adobe photoshop CS4 software (Adobe Systems Incorporated, San Jose, CA, USA). The results of open field test were statistically analyzed using one-way analysis of variance (ANOVA) by GraphPad Prism and the results for Barnes maze test were analyzed using repeated measures ANOVA using SPSS version19.0 (SPSS Inc., Chicago, IL, USA). The other results were statistically analyzed by two-way ANOVA followed by Tukey’s multiple comparison test. *P* < 0.05 was considered statistically significant.

## Results

### The Inhibition of RIPK1 by Nec-1 Attenuates Postoperative Cognitive Deficits in D-Gal-Induced Aged Mice

In the open field test, there were no obvious differences between control, Sur+DMSO group and Sur+Nec1 group 1 day after surgery (crossing number: *F*_(2,31)_ = 1.606, *P* = 0.216; time in central zone: *F*_(2,31)_ = 0.043, *P* = 0.958; Figure 1B). The analysis by repeated measures ANOVA showed in the Barnes maze test, the errors were statistical different among three groups (*F* = 5.553, *P* = 0.009). Specially, the errors of Sur+DMSO group were significantly more than that of the control and Sur+Nec1 groups on the 3rd day after surgery (*post hoc* test *P* = 0.016 vs. control; *P* = 0.039 vs. Sur+Nec1 group; Figure 1C). However, there was no statistical difference in the latency to the target hole among three groups (*P* > 0.05). These showed that Nec-1 attenuated the impairment of spatial learning and memory induced by surgery.

Problem solving, short-term memory and long-term memory are important elements of executive function (Zurek et al., 2016). Here we evaluated executive function by puzzle box test, where mice were presented with progressively more difficult tasks to reach the dark goal box. The latency to goal box during the first exposure to different tasks was used to assess problem solving ability. The latency to goal box during the retest 2 min and 24 h after the first exposure to the task was used for short-term memory and long-term memory, respectively. As shown in Figure 1D, the latency for the burrowing task was obviously longer than that of underpass task in each group (problem solving; *F*_(2,62)_ = 77.890, *P* < 0.001). No obvious difference of short-term memory was detected among three groups (*F*_(2,31)_ = 1.943, *P* = 0.160). However, using a two-way ANOVA analysis we found there was a significant effect for task (*F*_(1,31)_ = 22.730, *P* < 0.001) and groups (*F*_(2,31)_ = 12.560, *P* < 0.001), as well as task × group interaction (*F*_(2,31)_ = 3.382, *P* = 0.046) in long-term memory. Further analysis by Tukey’s multiple comparisons test found the mice in Sur+DMSO group spent more time to complete the underpass task tested 24 h after the first exposure than control and Sur+Nec1 group (*P* = 0.002 vs. control; *P* < 0.001 vs. Sur+Nec1 group), suggesting impairment of executive function and memory to simple low difficult task in aged mice exposed to surgery, which could be rescued by Nec-1 pretreatment.

### The Inhibition of RIPK1 by Nec-1 Alleviates Surgery-Induced Neuroinflammation in D-Gal-Induced Aged Mice

**Figure 2:**
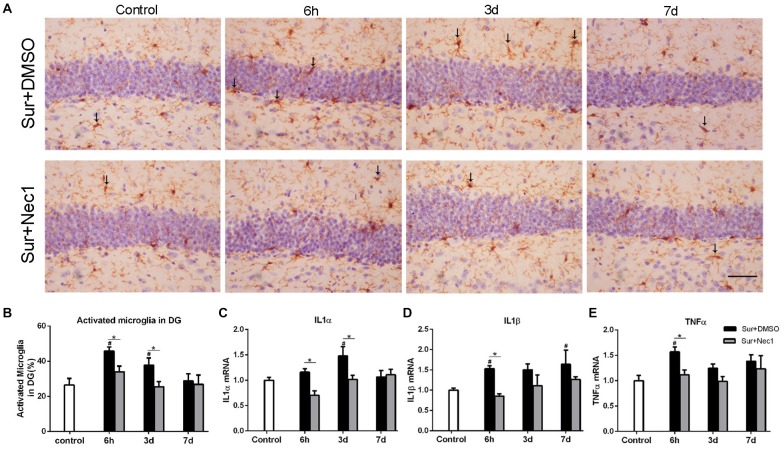
The inhibition of RIPK1 by Nec-1 alleviates surgery-induced neuroinflammation in D-Gal-induced aged mice. **(A)** Representative images of Iba-1 staining (yellow) in the dentate gyrus. The typical activated microglial cells labeled by Iba-1 staining were indicated with arrows. Bar = 50 μm. **(B)** The percentage of Iba-1 immunopositive activated microglia was counted in dentate gyrus. Panels **(C–E)** represent qPCR analysis of mRNA levels of inflammatory factors IL-1α, IL-1β and TNF-α in the hippocampus. Data were expressed as the mean ± SEM. ^#^*P* < 0.05, compared with control group; *P < 0.05, in comparison between Sur+DMSO and Sur+Nec1 groups by two-way ANOVA followed by Tukey’s multiple comparisons test (*n* = 6 per time point per group).

Activated microglia is characterized by bigger cell body and shortened or twisted branches, and is usually used to mark neuroinflammation (Zhang et al., 2017). In the study, compared to the control, microglia was obviously activated in the Sur+DMSO group at 6 h and 3 days after surgery (*P* = 0.007 at 6 h and *P* = 0.041 at 3 days), which was obviously limited at Sur+Nec1 group (*P* = 0.014 at 6 h; Figures 2A,B). Similarly, using a two-way ANOVA, we found that there was a significant effect for groups in the mRNA levels of inflammatory levels IL-1α, IL-1β and TNF-α (IL-1α: *F*_(2,45)_ = 6.300, *P* = 0.004; IL-1β: *F*_(2,45)_ = 9.140, *P* < 0.001; TNF-α: *F*_(2,45)_ = 8.513, *P* < 0.001). Further, Tukey’s multiple comparisons test revealed compared to control, the mRNA levels of IL-1α, IL-1β and TNF-α all were increased in the hippocampus of Sur+DMSO group after surgery (IL-1α: *P* = 0.012 at 3 days; IL-1β: *P* = 0.046 at 6 h; TNF-α: *P* = 0.005 at 6 h; Figures 2C–E). Compared to the Sur+DMSO group, the mRNA levels of IL-1α, IL-1β and TNF-α all were decreased in Sur+Nec1 group during the first 3 days after surgery (IL-1α: *P* = 0.005 at 6 h, *P* = 0.017 at 3 days; IL-1β: *P* = 0.016 at 6 h; TNF-α: *P* = 0.037 at 6 h; Figures 2C–E). These showed the inhibition of Nec-1 on the postoperative neuroinflammation.

### The Inhibition of RIPK1 by Nec-1 Decreases Surgery-Induced Necroptosis in D-Gal-Induced Aged Mice

**Figure 3:**
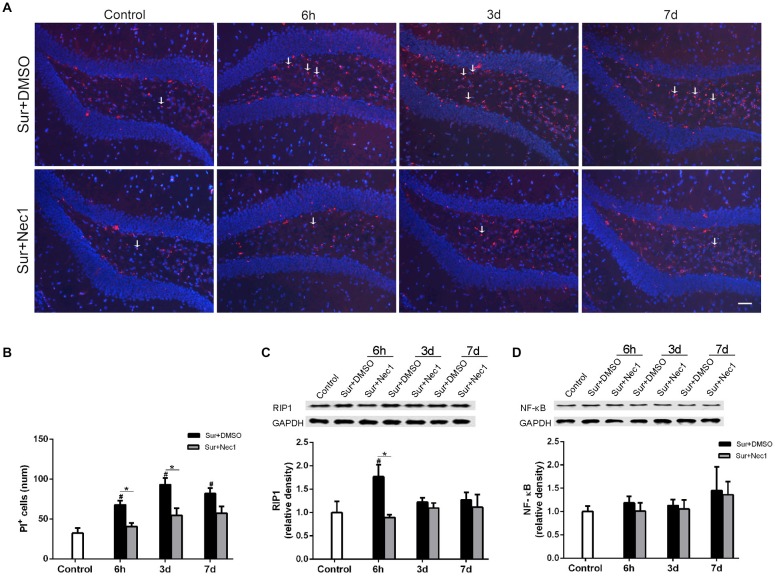
The inhibition of RIPK1 by Nec-1 decreases surgery-induced necroptosis in D-Gal-induced aged mice. **(A)** Representative images of propidium iodide (PI) staining (red) in the dentate gyrus. Nuclei were stained by 4′,6-diamidino-2-phenylindole (DAPI). The arrows indicated some typical cells stained by PI. Bar = 50 μm. **(B)** The PI-positive cells were counted in the DG. **(C,D)** Western blot analysis of RIP1 **(C)** and NF-κB **(D)** in the hippocampus. The upper panel showed the protein bands of RIP1 and NF-κB, and GAPDH was reference control. The lower panel showed that relative integrated density value of RIP1 or NF-KB to GAPDH. Data were expressed as the mean ± SEM. ^#^*P* < 0.05, compared with control group; *P < 0.05, in comparison between Sur+DMSO and Sur+Nec1 groups by two way ANOVA followed by Tukey’s multiple comparison test (*n* = 4 per time point per group).

PI is impermeable to the intact cell membrane and commonly used to label necrotic cell death (Kanno et al., 2015). As expected, only a few of PI-positive cells in dentate gyrus were detected in the control group (Figure 3A). Two-way ANOVA statistical analysis indicated that there were significant differences in the number of PI-positive cells among three groups (*F*_(2,27)_ = 37.13, *P* < 0.001). Compared to the control, the number of PI-positive cells were markedly increased in the Sur+DMSO group at 6 h, 3 days and 7 days after surgery with a peak at 3 days (*P* = 0.004 at 6 h; *P* < 0.001 at 3 days and 7 days), which was significantly inhibited by Nec-1 pretreatment (*P* = 0.027 at 6 h; *P* = 0.002 at 3 days; *P* = 0.047 at 7 days; Figures 3A,B). In addition, RIPK1 expression was also statistically different among the groups (*F*_(2,27)_ = 4.718, *P* = 0.018). Tukey’s *post hoc* test showed RIPK1 expression was increased in the Sur+DMSO group 6 h after surgery, relative to control and Sur+Nec1 group (*P* = 0.023 vs. control; *P* = 0.009 vs. Sur+Nec1 group; Figure 3C). RIP1 also mediates the activation of transcript factor NF-κB, then NF-κB drives the transcription of many classical pro-inflammatory cytokines, that will trigger inflammatory response (Silke et al., 2015). However, no obvious difference of NF-κB was detected between control, Sur+DMSO and Sur+Nec1 groups (*F*_(2,27)_ = 0.903, *P*_group_ = 0.417; *F*_(2,27)_ = 0.807, *P*_time_ = 0.456; Figure 3D).

### The Inhibition of RIPK1 by Nec-1 Ameliorates Surgery-Induced Synaptic Damage in D-Gal-Induced Aged Mice

**Figure 4:**
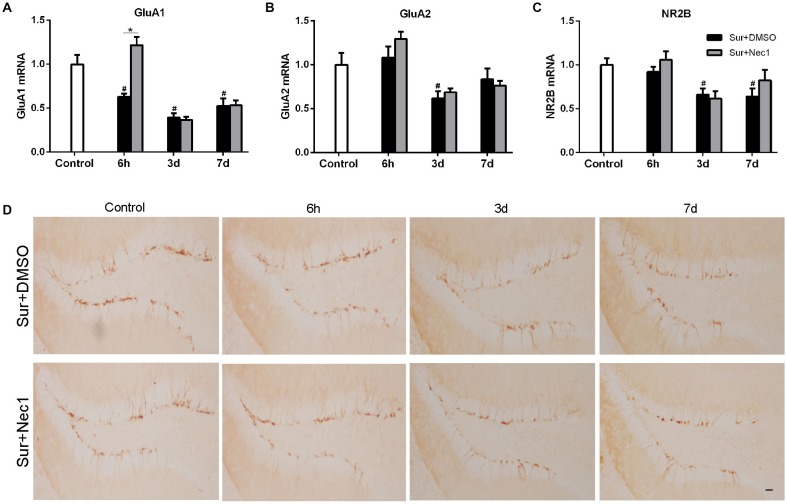
Nec-1 ameliorates surgery-induced synaptic damage in D-Gal-induced aged mice. **(A–C)** Represents qPCR assay for GluA1, GluA2 and NR2B, respectively. Data were expressed as the mean ± SEM (*n* = 6 per time point per group, two way ANOVA followed by Tukey’s multiple comparison test). ^#^*P* < 0.05, compared with control group; *P < 0.05, in comparison between Sur+DMSO and Sur+Nec1 groups. **(D)**. Representative images of Doublecortin (DCX) staining (yellow) in the dentate gyrus. Bar = 50 μm.

Next, we attempted to investigate the possible mechanism by which Nec-1 attenuated anesthesia and surgery-induced postoperative memory and cognitive impairment. NMDA receptors and AMPA receptors have been shown to be essential for synaptic plasticity, which is considered as the basis for spatial learning and memory formation (Hardingham and Bading, 2010; Zhang et al., 2013; Zhou et al., 2018). Therefore, we assessed the expression of AMPAR subunit GluA1 and GluA2 and NMDAR subunit NR2B level in hippocampus (Figures 4A–C). Two-way ANOVA analysis revealed that there was a significant effect for time (*F*_(2,45)_ = 15.880, *P* < 0.001) and groups (*F*_(2,45)_ = 26.940, *P* < 0.001), as well as time × group interaction (*F*_(4,45)_ = 8.429, *P* < 0.001) in GluA1 expression; there was a significant effect for time in GluA2 expression (*F*_(2,45)_ = 8.687, *P* < 0.001; *F*_(2,45)_ = 7.261, *P* = 0.002), but not for groups; and there was a significant effect for groups (*F*_(2,45)_ = 7.261, *P* = 0.002) and time (*F*_(2,45)_ = 6.255, *P* = 0.004) in NR2B expression. Further, Tukey’s multiple comparison test showed that GluA1, GluA2 and NR2B mRNA expression were dramatically decreased in hippocampus after surgery (vs. control: GluA1: *P* < 0.006 at 6 h, *P* < 0.001 at 3 days, *P* = 0.004 at 7 days; GluA2: *P* = 0.040 at 3 days; NR2B: *P* = 0.018 at 3 days, *P* = 0.011 at 7 days). Intriguingly, Nec-1 pretreatment significantly suppressed the loss of GluA1 at 6 h after surgery (vs. Sur+DMSO group, *P* < 0.001). These suggested corresponding to cognitive decline, surgery induced a progressive loss in hippocampal GluA1, GluA2 and NR2B expression in aged mice, while Nec-1 prior to surgery prevented synaptic GluA1 loss.

Doublecortin (DCX), an immature neuronal marker, was used to detect the neurogenesis in dentate gyrus which plays a key role in spatial memory function (Hem et al., 2016). Hence, we also examined the possibility of POCD in aged mice might be related to a deficiency in dentate gyrus neurogenesis by DCX immunostaining. But our results showed that anesthesia and left partial hepatectomy did not trigger impairment of neurogenesis in dentate gyrus in aged mice (Figure 4D).

## Discussion

Our aim was to investigate whether inhibiting RIPK1 by Nec-1 could limit neuroinflammation and attenuate postoperative cognitive deficits in D-Gal-induced aged mice. We showed that anesthesia and surgery induced a significant deficit in spatial memory acquisition and long term memory to execute simple low difficult task in D-Gal-induced aged mice. Inhibiting RIPK1 by Nec-1 strikingly alleviated postoperative cognitive impairment and amplified neuroinflammation, necroptosis and GluA1 loss in hippocampus. These suggest that targeting RIPK1 by Nec-1 may serve as a promising therapeutics for prevention of POCD in elderly patients.

Accumulating evidences have been demonstrated that neuroinflammation provoked by anesthesia and surgery is the critical mechanism in the pathogenesis of POCD (Skvarc et al., 2018). Limiting perioperative neuroinflammation obviously alleviates POCD and pathological impairment of brain after surgery (Ma et al., 2015; Sun et al., 2017). However it is a challenging work to limit sterile neuroinflammation during perioperative period due to the blood-brain barrier and the lack of molecular target and specific drugs. RIPK1 is a key molecule switching the NF-κB-dependent inflammatory response, the caspase-8-dependent apoptosis and the MLKL-dependent necroptosis (Pasparakis and Vandenabeele, 2015; Silke et al., 2015). Recent studies found RIPK1 modulated the neuroinflammation in Alzheimer’s disease (Caccamo et al., 2017; Ofengeim et al., 2017). These suggest RIPK1 is a possible target for limiting sterile neuroinflammation during POCD. Nec-1, a RIPK1 specific inhibitor can easily enter the brain by crossing the impaired blood brain barrier (Yang et al., 2017). So we tried to detect whether inhibiting RIPK1 by Nec-1 could limit neuroinflammation and alleviate postoperative cognitive deficits. As expected, anesthesia and surgery induced a significant deficit in spatial memory acquisition and long term memory to execute simple task and obvious neuroinflammation in D-Gal-induced aged mice (Figures 1, 1, 2). Inhibiting RIPK1 by Nec-1 strikingly alleviated postoperative cognitive deficits and neuroinflammation (Figures 1, 1, 2). Further study on mechanism found that anesthesia and surgery significantly increased the number of PI-positive cells and the expression of RIPK1, but not the expression of NF-κB in hippocampus, suggesting increased necrotic cell death (Figure 3). And inhibiting RIPK1 by Nec-1 dramatically decreased necrotic cell death after surgery (Figure 3). Previous study showed that necrotic cell could trigger sterile inflammatory responses (Pasparakis and Vandenabeele, 2015; Silke et al., 2015). Thus during POCD, inhibiting RIPK1 by Nec-1 limited neuroinflammation mainly by decreasing necrotic cell death after surgery.

An important feature of neuroinflammation induced by anesthesia and surgery is age-dependent (Le et al., 2014; Xu et al., 2014). For example, our previous study showed that surgery significantly activated microglia and increased the levels of TNF-α and IL-1β in aged rats, but not in adult rats (Le et al., 2014). However the reason for the age-dependent feature of postoperative neuroinflammation remains unclear. In the study, we found obvious necrotic cell death in aged mice after surgery (Figure 3) and inhibiting RIPK1 by Nec-1 significantly limited inflammatory gene expression. We also detected no obvious alteration of NF-κB in hippocampus after surgery. It is well-documented that RIPK1–RIPK3–MLKL-mediated necroptosis causes severe inflammation through releasing cellular damage-associated molecular patterns (DAMPs) such as IL-1α, IL-1β and HMGB1 (Pasparakis and Vandenabeele, 2015; Silke et al., 2015). Thus the postoperative necroptosis in aged brain might be a reason of strong and persistent neuroinflammation in aged individuals after surgery.

Our current study is the first to reveal the role of RIPK1-mediated necroptotic signaling in postoperative cognition impairment and neuroinflammation. However, there are some limitations in this study. Firstly, we just detected only a single dose of Nec-1 and once per mice. The optimal administration approach of Nec-1 remains unclear. Secondly, Nec-1 can regulate signaling complex containing members of RIPK family, so further in-depth study need focus on RIP1-RIP3 interaction and complex connection between RIPK1-regulated necroptosis and inflammation, to identify the detailed molecular targets that mediate protection by Nec-1.

## Author Contributions

SD conducted aged modeling, hepatectomy modeling and qPCR. XW performed tissue preparation and western blot. CQ and GC participated in behavioral tests and immunohistochemistry. SQ performed data processing and statistical analysis. JT secured funding acquisition and took overall responsibility for the study. JT, SQ and SD designed the experiment, drafted and revised the manuscript.

## Conflict of Interest Statement

The authors declare that the research was conducted in the absence of any commercial or financial relationships that could be construed as a potential conflict of interest.
